# Evidence of man-vector contact in torn long-lasting insecticide-treated nets

**DOI:** 10.1186/1471-2458-13-751

**Published:** 2013-08-14

**Authors:** Virgile Gnanguenon, Roseric Azondekon, Frederic Oke-Agbo, Arthur Sovi, Razaki Ossè, Gil Padonou, Rock Aïkpon, Martin C Akogbeto

**Affiliations:** 1 Centre de Recherche Entomologique de Cotonou (CREC), Cotonou, Benin; 2 Faculte des Sciences et Techniques de l’Université d’Abomey-Calavi, Abomey-Calavi, Benin; 3 University of Massachusetts Amherst, Amherst, USA

**Keywords:** Long-lasting insecticidal nets, Man-vector contact, *Anopheles gambiae*

## Abstract

**Background:**

Studies indicate that physical damage to long-lasting insecticide-treated nets (LLINs) occurs at a surprisingly rapid rate following net distribution. To what extent does such damage affect the impact of LLINs? Can vectors pass a compromised LLIN barrier to bite? Do more resistant vectors enter the insecticide-treated nets (ITNs) through holes?

**Methods:**

The study was carried out in three geo-locations. Two types of LLINs (polyester and polyethylene) with ‘standardized’ physical damage were compared with similarly damaged, but non-insecticidal (control) nets. The proportionate Holes Index (pHI) of each net was 276. Mosquitoes were captured inside the nets, identified taxonomically, and subjected to molecular analysis to estimate *Knock-down resistance* (*Kdr*) frequency.

**Results:**

The most commonly observed species was *Anopheles gambiae,* accounting for approximately 70% (1,076/1,550) of the total mosquitoes collected both in LLINs and non-insecticidal nets. When compared with controls, number of vectors captured in torn LLINs was significantly reduced. Nonetheless in a night, an average of 5 *An. gambiae s.l* could enter the damaged LLINs to bite. Similar numbers of resistant mosquitoes were collected in both LLINs and non-insecticidal (control) nets (p > 0.05).

**Conclusions:**

At a pHI of 276, man-vector contact was observed in torn LLINs. The insecticide at the surface of LLINs could only reduce the number of vectors. Resistant mosquitoes have opportunity to enter both non-insecticidal (control) nets and LLINs to bite.

## Background

Long-Lasting Insecticide-Treated Nets (LLINs) are widely used in sub-Saharan Africa for malaria vector control [1-3]. This technology is based on the slow release of pyrethroid insecticides, rendering them wash-resistant and extending insecticide residual effectiveness to at least three years without the need of re-treatment [4]. That is why approximately 289 million LLINs were distributed in Africa between 2008–2010 [5]. The initial impact of this intervention is thought to be significant [6-8]. In fact, LLINs can reduce man-vector contact by providing a physical barrier between the human sleeping under the LLIN and the malaria vector mosquito. This protection is enhanced by an insecticide (chemical barrier) that deters, repels, or kills vectors that attempt to bite the sleeper. LLIN that has its physical barrier (mesh) intact will rarely allow mosquitoes to reach the person sleeping under the LLIN [9]. But the critical question about the real operational life (physical integrity and bio-efficacy) of LLINs remains. Current LLIN distributions and replacement needs are based on an assumption that LLINs have an average useful life of 3–5 years [10]. But, there is a significant lack of research in LLIN survival time and variation in performance between LLINs of different textiles. To solve this deficit, the Vector Control Working Group of the Roll Back Malaria (RBM) Partnership called for more field research on LLIN durability [11].

To answer this call, a retrospective study on LLIN durability was conducted in Benin in 2010 [12]. This study showed losses associated with LLIN durability within 12–25 months after a net distribution by the National Malaria Control Program (NMCP) in 2008–2009. A substantial proportion of the LLINs (32%) had finger-sized holes, and/or fist-sized (10%) holes. Other studies conducted in Uganda on physical integrity of mosquito nets showed that 45%-78% of the nets were damaged even within a year of use in operational conditions [13,14]. Recent LLIN assessments in Kenya and Benin, reported a faster-than-anticipated loss of physical (durability) and insecticidal integrity (bio-efficacy), raising concerns about the duration of LLIN effective life [15,16]. In another study [17], repeated treatment of Insecticide Treated Nets (ITNs) with holes protected humans from mosquito bites in a vector susceptible area but failed to do so in a vector resistant area.

Other published studies conducted in Kenya [18,19] on ITNs durability found the majority of ITNs assessed in operational use with holes (78%-99.5%). Using different cut-offs of the proportionate Holes Index (pHI), around half of the torn ITNs (50.5%-61.3%) were classified as ‘effective nets’ and the other one as ‘ineffective nets’.

These observations raise the questions: Can vectors pass a compromised LLIN barrier to bite? Does the insecticidal feature of LLIN do a better job compensating for physical damage than was the case with the ITN? Or do users of torn LLINs remain protected by the insecticidal effect, as suggested in the literature [20]. Finally, do more resistant vectors enter the LLINs through holes?

## Methods

### Study design

To estimate the presence of malaria vector in the torn LLINs and to assess the potential of insecticidal barrier to reduce mosquito entry rate under such conditions, mosquitoes were caught inside torn LLINs and similarly damaged, but non-insecticidal, nets (controls) at three geo-locations for a period of 5 months (April to August) in 2010. Vector density inside the nets was quantified and a comparison was made between the LLINs with holes and the control (non-insecticidal net in a similar condition).

### Study areas and selection of households

**Figure 1:**
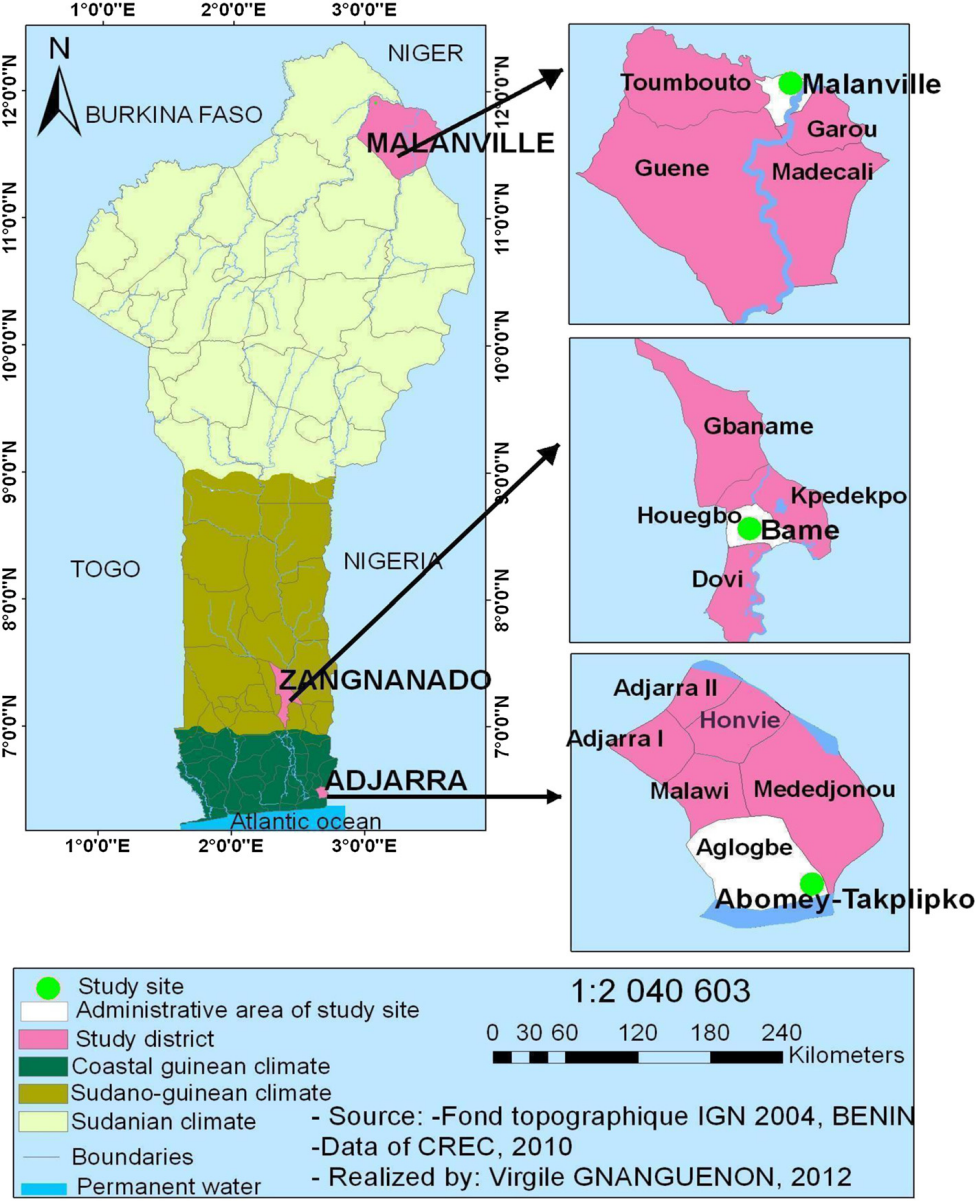
Study area.

There were three study locations each of which had permanent vector breeding sites giving rise to a feature of relatively high vector densities at night, including the nights that vector collections were made. The locations were: Abomey-Takplikpo (6°31′54″N and 2°39′56″E) in Adjarra district, and Bame (7°16′46″N and 2°24′46″E) in Zangnanado district. Abomey-Takplikpo is located in a Guinean-bioclimatic zone and Bame is located in an intermediate bioclimatic zone (tropical Sudano-Guinean climate) (Figure 1). The third location was in the North, in a peri-urban area of Malanville district (11°50′27″N and 3°24′08″E), which has a Sudanian semi-arid bioclimatic zone. The Guinean-bioclimatic zone is located in the south, near the Atlantic coast with two rainy seasons (April–July and September–November) and an average annual rainfall of >1500 mm with degraded tropical forest. The Sudano-Guinean climate zone is located in the center with an average rainfall of 1000 mm per year, characterized as humid savanna. The Sudanian semi-arid bioclimatic zone is located in the north, with only one rainy season from June to October (mean annual rainfall below 900 mm) and characterized by a dry savanna. The three geo-locations are known to have no significant differences in mosquito composition. Resistance levels of vectors to insecticides at these sites were also known. In the Guinean and Sudano-Guinean sites, vectors were resistant, whereas in the Sudanese site, vectors were susceptible to pyrethroid insecticides [17,21,22].

Six random houses whose owners had signed a written consent to participate in the study were selected at each site. The criteria for selection were that each house contains a sleeping room with a close-fitting door and a window. The points of entries for mosquitoes were through open doors or eave gaps between walls and roofs. At the end of the study, participating households received new LLINs.

### Mosquito nets

The study evaluated two types of LLINs most commonly distributed and found in the market place in Benin: polyester 75D (PET-75D) (Permanet® 2.0 Vestergaard Frandsen SA, Aarhus, Denmark), and polyethylene 150D (PE-150D) (Olyset®, Sumitomo Chemicals, Osaka, Japan). Results were compared with regard to a non-insecticidal net (manufacturer: Palutech Benin, Thailand). The PET-75D and PE-150D LLINs were blue in color and the control nets, white. The colors are the ones most commonly found in the market place, and communities surrounding the study locations. Mosquito preference for a particular colored net is not known and there was a limitation in this study design of not being able to separate possible confounding factors due to variation in blue colored nets attractiveness from that of white. PET-75D LLINs were treated with deltamethrin (55 mg/m^2^) and dimensions were 1.95 m long × 1.6 m wide × 2.0 m high. PE-150D nets, had permethrin (2%) incorporated into the net fiber (polyethylene resin) and they were 1.8 m long × 1.9 m wide × 1.5 m high. The non-insecticidal nets had polyester fiber and were 2 m long × 1.6 m wide × 1.9 m high.

**Figure 2:**
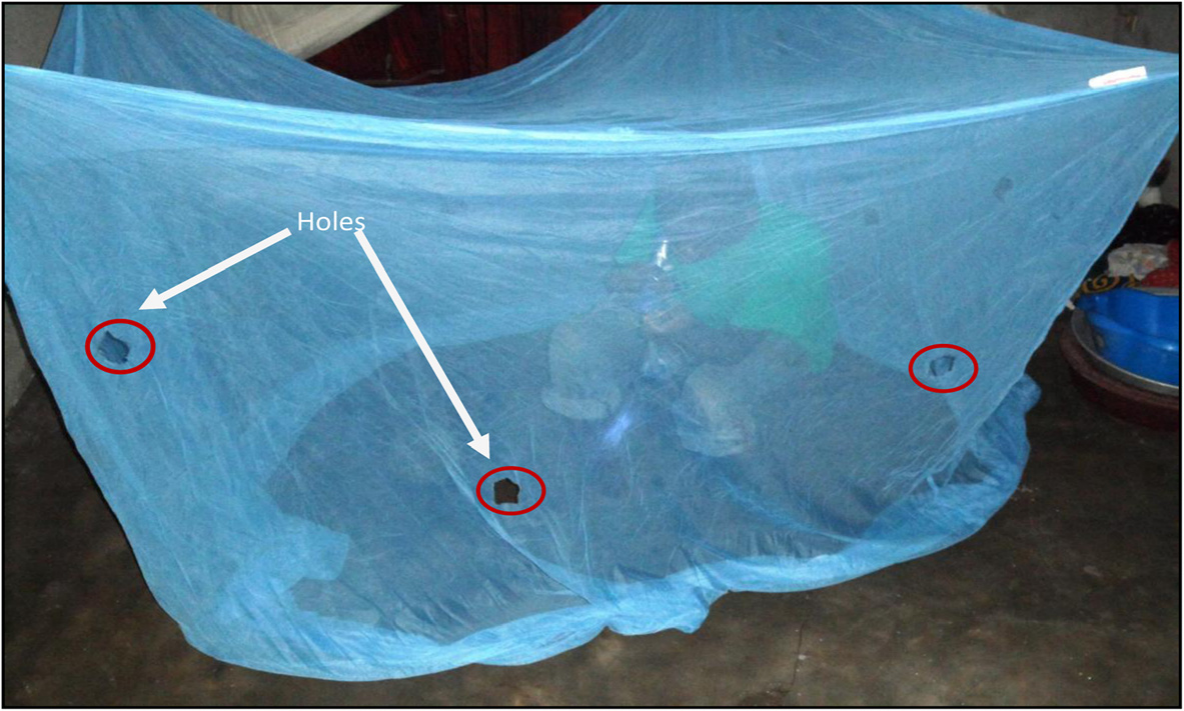
Photo of a net with holes and collector.

To simulate torn nets and assess chemical barrier without need of retreatment by dipping, 12 square holes of 4 cm × 4 cm each (3 holes by side were cut at the same standardized positions except in the roof) in all three types of nets. According to the method described by World Health Organisation [23], the pHI of each net was 276 (Figure 2).

### Collectors and mosquito sampling in the torn nets

Comparison testing of the three nets: PET-75D, PE-150D and “Control” occurred concurrently twice a month for five months (April to August 2010) in the three study locations. Two PE-150D, two PET-75D and two non-insecticidal nets were hung (at the habitual sleeping place but not over bed) in the six selected houses at each site. The nets with standardized damage as well as the collectors were randomly rotated between the participating households on successive nights to adjust for any variation in attractiveness to mosquitoes.

Mosquito collectors, stationed under the nets, were selected using three criteria: Each collector (1) was an adult volunteer, (2) signed a written consent to participate in the study, and (3) had access to no other mosquito net. Collectors were trained to capture mosquitoes using an aspirator and a flashlight while inside the nets (Figure 2). They were informed 24 hours before each collection night and slept in the day and tried to satisfy natural call before starting the collection.

The collections were done continuously through the night from 9:00 p.m to 5:00 a.m and volunteers did not exit the nets during the night (to avoid possibility of creating a gap while exiting and re-entering the nets during collections). Mosquitoes were caught as soon as they entered the nets before having possibility to feed on the collectors. Human Biting Rate (HBR) was defined here as: “the number of mosquitoes caught per collector while in the net”.

No other household member was present in the house during the collections (in order not to attract vector from the nets and avoid exposure to vector bite).

### Measuring mosquito diversity in the torn nets

All captured mosquitoes were identified to species using taxonomic keys of Gillies & De Meillon [24] and Gillies & Coetzie [25]. We determined the species richness (S) associated with each type of net at each location using the Shannon’s diversity index, H, defined as:

$$H=\sum -\left(P_{i}*{\ln\;P}_{i}\right)$$

i = 1

Where:

H = the Shannon diversity index

P_i_ = fraction of the entire population made up of species i

S (Richness) = numbers of species encountered

∑ = sum from species 1 to species S

The H value indicates the number of species, as well as their relative abundance compared with the others in the collections [26].

### Frequency of *Kdr* mutations in *An. gambiae s.l* collected in the LLINs

Mosquitoes were tested using polymerase chain reaction-restriction fragment length polymorphism (PCR-RFLP) analyses to detect the presence of *Knock down resistance (Kdr)* genes [27]. The mosquitoes tested were selected from the total pool as follow:

–Around 20% of the total of *An. gambiae s.l* was randomly selected from each site in the resistance area.

–In the susceptible area, 40% of *An. gambiae s.l* collected was randomly selected to have enough data to compare the presence of resistant and susceptible genotypes in holed nets in this area.

### Data analysis

Diversity data were analyzed using PAST 2.07, a diversity software package. Comparison of the Shannon diversities observed for the LLINs versus the non-insecticidal (control) net collections was made using Student’s t test. We assessed the effect of LLINs on mosquito density using the Incidence of Density Ratio (IDR) of LLINs versus non-insecticidal (control). Logistic regression model and odds ratio were used to assess variability of *Kdr* frequency between LLINs and non-insecticidal nets and to check if more resistant mosquitoes enter LLINs compared to the non-insecticidal nets. Mosquito collections were done concurrently with the three types of nets at each site and the variable “resistance level of the location” was not included in the data analysis.

Significant differences were those with a p-value less than or equal to 0.05. These tests were conducted using R 2.14.1 software.

### Ethical considerations

This study was approved by the ethical committee of the Ministry of Health. Community leaders were briefed on the protocol before the study and gave verbal consent before the study began. Written consent was obtained from all participating volunteers, who were vaccinated against yellow fever and provided with malaria prevention and curative treatments according to World Health Organization (WHO) recommended regimen (on the basis of fever and detectable *P. falciparum* parasitemia).

## Results

### Diversity and geo-variability of mosquitoes collected in the damaged nets

A total of 10 species belonging to five genera (*Culex*, *Aedes*, *Coquillettidia*, *Mansonia* and *Anopheles*) were collected inside the nets with standardized physical damage (Table 1). There were four species of *Culex,* three species of *Anopheles*, and one species, each, of *Mansonia, Aedes,* and Coquillettidia. There was more species in the non-insecticidal net collections (S = 10), than in the LLIN collections, S = 7 for PET-75D collections and S = 6 for PE-150D collections.

**Table 1:** Mosquito diversity (S) in collections from damaged bed nets: non-insecticidal (control) nets versus PET-75D and PE-150D LLINs

**Species**	**Non-insecticidal nets**			**PET-75D**			**PE-150D**		
	**ni**	**pi**	**(p**_**i**_**) ln (pi)**	**ni**	**pi**	**(n**_**i**_**) ln (n**_**i**_**/N)**	**ni**	**pi**	**(n**_**i**_**) ln (n**_**i**_**/N)**
***Anopheles gambiae***	553	0.66	−0.27	234	0.62	−0.30	289	0.87	−0.12
***Anopheles pharoensis***	36	0.04	−0.14	38	0.10	−0.23	12	0.04	−0.12
***Anopheles ziemanni***	31	0.04	−0.12	10	0.03	−0.10	4	0.01	−0.05
***Aedes aegypti***	1	0.00	−0.01	0	0	NA	0	0	NA
***Culex. quinquefasciatus***	72	0.09	−0.21	30	0.08	−0.20	12	0.04	−0.12
***Culex gr decens***	14	0.02	−0.07	18	0.05	−0.15	1	0.00	−0.02
***Culex nebulosus***	1	0.00	−0.01	3	0.01	−0.04	0	0	NA
***Culex annulioris***	2	0.00	−0.01	0	0	NA	0	0	NA
***Coquillettidia Cristata***	1	0.00	−0.01	0	0	NA	0	0	NA
***Mansonia africana***	123	0.15	−0.28	44	0.12	−0.25	15	0.05	−0.14
**Richness (S)**	10			7			6		
**Shannon-wiener index (H) with 95% CI**	1.13[0.99-1.14]			1.25[1.14-1.34]			0.57[0.44-0.67]		
**T scores**	NA			−1.87			−7.70		
**P-values**	NA			0.06			0.000		

*N* total number of individuals, *ni* number of individuals found in the ith species, *pi* proportion of individuals found in the ith species, *ln* the natural (Naperian) logarithms, *NA* Not Applicable.

There was a highly significant difference in diversity between PE-150D - and non-insecticidal net- collections in the same location (p < 0.001) (Table 1). PET-75D and non-insecticidal nets, showed no difference (p = 0.06). *An. gambiae s.l* accounted for approximately 70% of the collections (1,076/ 1,550).

**Figure 3:**
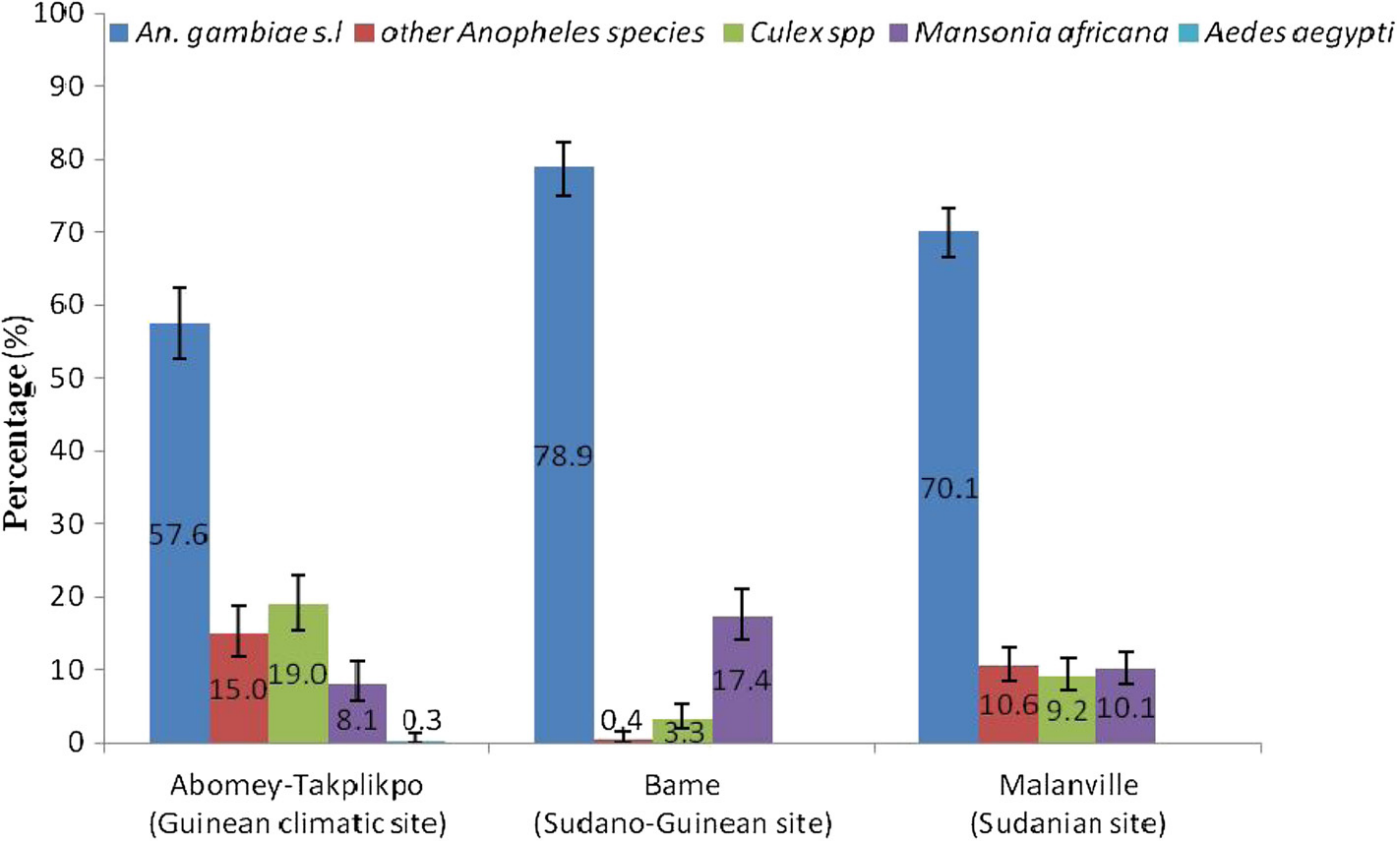
Proportion of mosquitoes collected in the nets at each geo-location.

The abundance of each mosquito species collected varied by location (Figure 3). There were 234 (57.6%) *An. gambiae s.l,* 61 (15.0%) other *Anopheles* species (*An. pharoensis* and *An. ziemanni*), 77 (19%) *Culex* spp, 33 (8*.*1%) *Mansonia africana,* and 1 (0.3%) *Aedes aegypti* collected at the Guinean climatic site*.* In contrast there were 362 (78.9%) *An. gambiae s.l,* 2 (0.4%) other *Anopheles* species (*An. pharoensis* and *An. ziemanni*), 15 (3.3%) *Culex* spp., and 80 (17.4%) *Mansonia africana* at the Sudano-Guinean site. The greatest number of mosquitoes was recorded at the sudanian site where 480 (70.1%) *An. gambiae s.l*, 73 (10.6%), other *Anopheles* species (*An. pharoensis* and *An. ziemanni*) 63 (9.2%) *Culex* spp., and 69 (10.1%) *Mansonia africana*. There was a high variability of *An. gambiae s.l* density in the torn nets between the bioclimatic areas (D = 198.447; df = 2; p < 0.001).

No feeding rate was recorded in this study because of the unfed status of the collected mosquitoes.

### Efficacy of damaged LLINs versus non-insecticidal nets in man-vector contact reduction

Densities of *An. gambiae s.l* were higher in the non-insecticidal nets than in the LLINs, regardless of the collection areas (Table 2). At Abomey-Takplikpo in the Guinean area, the Incidence Density Ratio (IDR) for *An. gambiae s.l* was significantly lower in the treated nets than in the non-insecticidal nets (PE-150D: IDR 0.31; 95% CI 0.22-0.43; p < 0.001; PET-75D: IDR 0.36; 95% CI 0.26-0.50; p < 0.001). The overall preventive effect against entry of *An. gambiae s.l* was 69% (95% CI 57%-78%) for PE-150D nets and 64% (95% CI 50%-74%) for PET-75D. In the Sudano-guinean (Bame) and Sudanian (Malanville) sites, the IDR were also lower in PET-75D and PE-150D nets than in the non-insecticidal nets (Table 2). The overall preventive effect against entry of *An. gambiae s.l* in the Sudano-Guinean site was 27% (95% CI 07%-42%) for PE-150D nets and 47% (95% CI 31%-59%) for PET-75D nets. In the Sudanian site, the overall preventive effect against entry of *An. gambiae s.l* was 49% (95% CI 37%-59%) for PE-150D nets and 61% (95% CI 51%-69%) for PET-75D nets. The IDR of PET-75D and PE-150D nets indicated that these LLINs provided similar levels of protection against malaria vectors.

**Table 2:** Prevention of mosquito entry into torn LLIN

**Areas**	**Species**	**Mosquito nets**	**Density**	**IDR (95% CI)**	**p-value**
**Abomey-Takplikpo (Guinean area)**	*An. gambiae*	Non-insecticidal nets	140	1	
		PE-150D	43	0.31 [0.22-0.43]	<0.001
		PET-75D	51	0.36 [0.26-0.50]	<0.001
	Other culicidae	Non-insecticidal nets	78	1	
		PE-150D	8	0.10 [0.05-0.21]	<0.001
		PET-75D	86	1.10 [0.81-1.50]	0.532
**Bame (Sudano-Guinean area)**	*An. gambiae*	Non-insecticidal nets	160	1	
		PE-150D	117	0.73 [0.58-0.93]	<0.001
		PET-75D	85	0.53 [0.41-0.69]	<0.001
	Other culicidae	Non-insecticidal nets	77	1	
		PE-150D	8	0.11 [0.05-0.22]	<0.001
		PET-75D	12	0.16 [0.08-0.29]	<0.001
**Malanville (Sudanian area)**	*An. gambiae*	Non-insecticidal nets	253	1	
		PE-150D	129	0.51 [0.41-0.63]	<0.001
		PET-75D	98	0.39 [0.31-0.49]	<0.001
	Other culicidae	Non-insecticidal nets	127	1	
		PE-150D	33	0.26 [0.18-0.38]	<0.001
		PET-75D	45	0.35 [0.25-0.50]	<0.001

For other mosquito species, IDR of treated nets were also lower than in the controls. The overall preventive effect against entry of other mosquito species was 90% (95% CI 79%-95%) for PE-150D nets but many other mosquito species were collected in the torn PET-75D nets compared with the non-insecticidal nets at the Guinean site (Table 2). At the Sudano-Guinean site, the overall preventive effect was 89% (95% CI 78%-95%) for PE-150D nets and 84% (95% CI 50%-74%) for PET-75D nets. At the Sudanian site, the overall preventive effect against entry of other mosquitoes was 74% (95% CI 62%-81%) for PE-150D nets and 65% (95% CI 50%-75%) for PET-75D nets. Globally, the two types of LLINs reduced other mosquito species entry at the same level.

**Figure 4:**
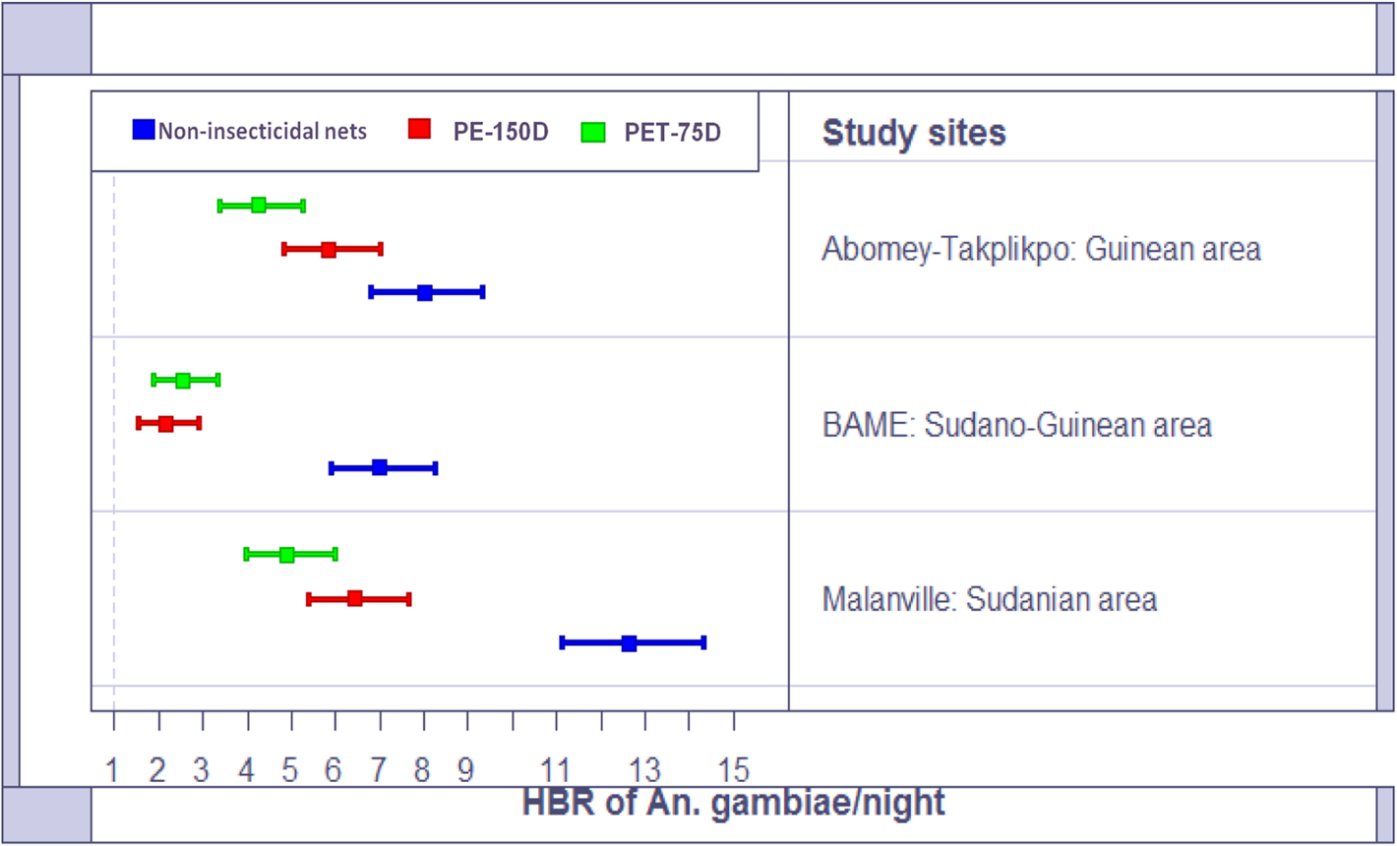
**Human biting rate of *An. gambiae s.l *per night per torn nets.**

**Figure 5:**
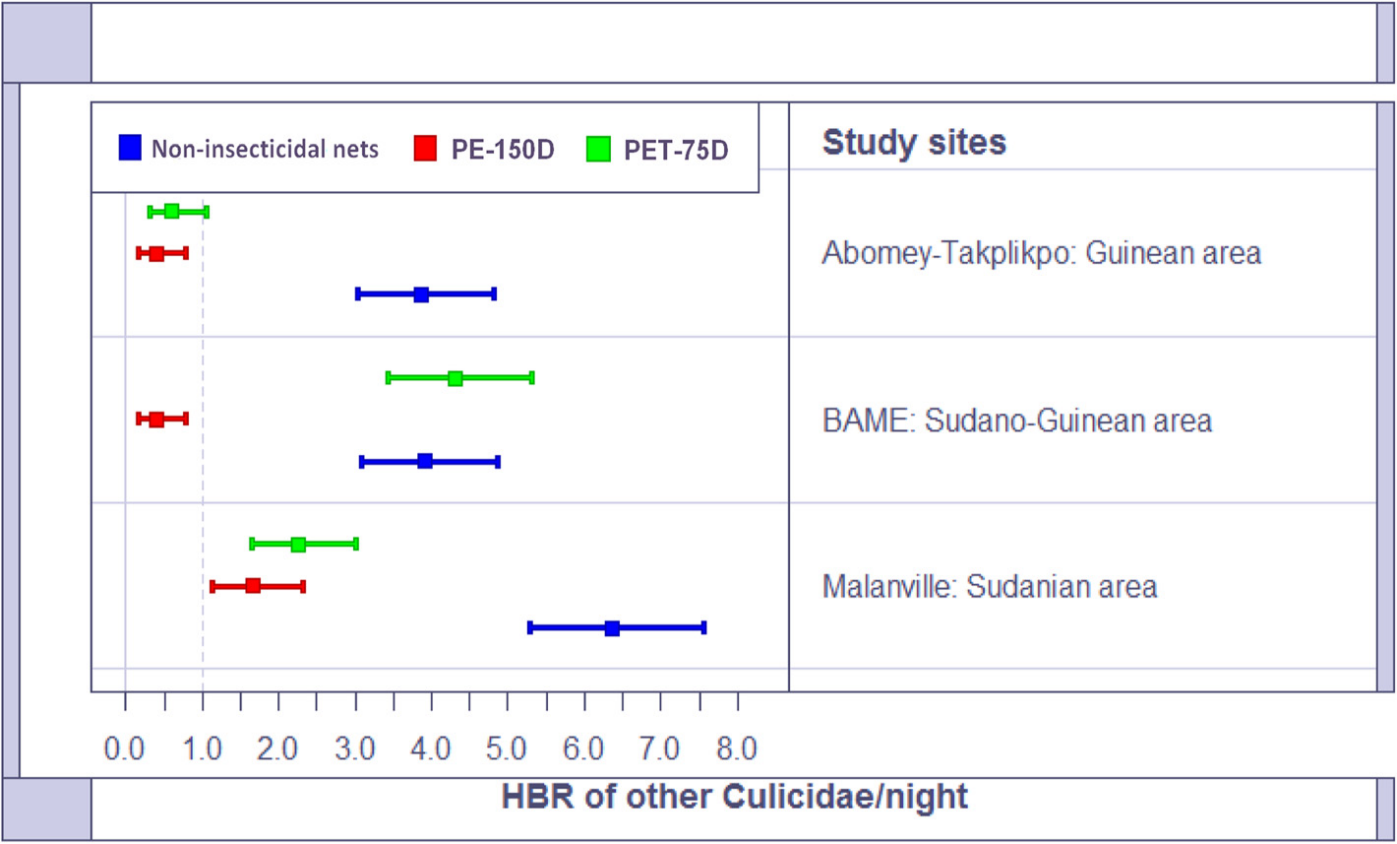
Human biting rate of other nuisant mosquito species per night per torn nets.

In summary, the average Human Biting Rate (HBR) of *An. gambiae s.l* was 12.65[11.13-14.32] in the non-insecticidal nets versus 6.45[05.38-07.67] for PET-75D LLINs and 4.9[03.97-05.97] for PE-150D LLINs in the Sudanian area (Figure 4). In Sudano-Guinean and Guinean sites, the average human biting rate of *An. gambiae s.l* was 7.5 (range 7–8) in a non-insecticidal net against 2.15 and 4.25 for PET-75D LLIN and PE-150D LLIN. The same observation was made with other mosquito species (Figure 5). 4–6 other species can bite a man in the non-insecticidal net in a night versus 1–4 for PET-75D and 1–2 for PE-150D LLINs.

### *Kdr* genotypic distribution of *Anopheles gambiae* caught in the torn LLINs

A total of 320 *Anopheles gambiae s.l* females were tested for *Kdr*. *Kdr* frequency was ranged from 0.66 to 0.81 in the females collected from the non-insecticidal (control) nets versus 0.66 to 0.94 in the LLIN collections (Table 3). No significant difference was observed between the *Kdr* frequencies observed in LLINs versus non-insecticidal nets (p > 0.05). In the susceptible area, no difference was observed between resistant and susceptible vectors collected. The *Kdr* frequencies recorded were also high in this area and not significantly different from the frequencies observed in the resistance area (Table 3). In the susceptible area, resistant and susceptible mosquitoes can enter the torn nets at similar level (p > 0.05).

**Table 3:** ***Kd*r and genotype frequencies of *Anopheles gambiae s.l *collected in torn nets**

**Area**	**Localities**	**Mosquito nets**	**SS**	**RS**	**RR**	**F ( *Kdr *)**	**OR (95% CI)**	**P-value**
**Resistance area**	**Abomey-Takplikpo (Guinean area)**	Non-insecticidal nets	2	7	15	0.77	1	
		PE-150D	0	3	8	0.86	1.88 [0.47-7.57]	0.37
		PET-75D	0	4	7	0.82	1.34 [0.37-4.79]	0.65
	**Bame (Sudano-Guinean area)**	Non-insecticidal nets	0	11	18	0.81	1	
		PE-150D	0	4	23	0.92	2.93 [0.87-9.83]	0.08
		PET-75D	0	3	24	0.94	3.98 [1.05-5.14]	0.04
**Susceptible area**	**Malanville (Sudanian area)**	Non-insecticidal nets	14	35	45	0.66	1	
		PE-150D	6	14	26	0.72	1.28 [0.74-2.21]	0.37
		PET-75D	12	11	28	0.66	0.96 [0.58-1.60]	0.89

*SS* homozygous susceptible; *RS* hybrid resistant and susceptible; *RR* homozygous resistant.

**Figure 6:**
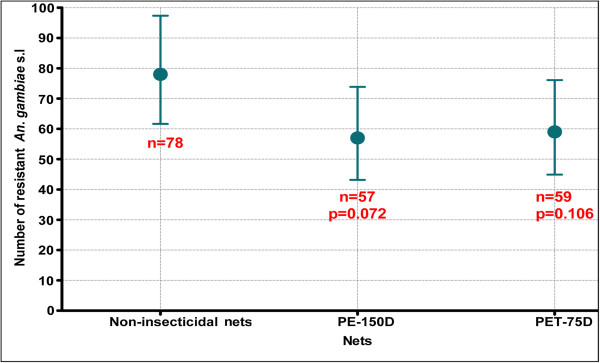
**Comparison of resistant *An. gambiae s.l. *entering LLIN versus non-insecticidal net.**

When we compared the total number of resistant females (RR) in the torn LLINs versus the controls, no significant difference was observed (p > 0.05) (Figure 6). This indicated that resistant mosquitoes can enter both LLINs and non-insecticidal nets at the same level and excluded the possibility to suspect a genetic cost related to the ability of resistant mosquitoes to enter non-insecticidal nets.

## Discussion

Mosquito diversity, observed in torn mosquito nets, could be useful in forecasting infection risk for malaria as well as other vector-borne diseases (chikungunya, filariasis, dengue, etc.). When LLINs were damaged and then tested, ten different mosquito species were caught in the mosquito nets. Four of the species were known as vectors of medical importance in Africa. *An. gambiae s.l* is the major malaria vector in Benin [21]. *Culex quinquefaciatus* transmits *Bancroftian filariasis* and West Nile Virus [28,29]. *An. pharoensis* and *Mansonia africana* are key vectors in transmitting Rift Valley Fever virus [30,31]. However, there was a significant difference in term of mosquito diversity between PE-150D LLINs and non-insecticidal (control) nets. No difference in mosquito diversity was observed between PET-75D LLINs and non-insecticidal (control) nets. But difference could be observed with a large sample size. These results suggested that the insecticide effect of LLINs could reduce the number of mosquito species that could enter torn nets.

The relative abundance of each mosquito species in the collections and by geo-location is more difficult to interpret since it may reflect the influence of various factors including the sampling method which targeted only endophilic species. Previous studies on mosquito species composition [32,33] using human landing catch and larvae collection have reported three times more mosquito species (28 species). But those collections were not conducted in the same assessment areas as this study. The few numbers of species collected in this study could not indicate that some species are better in entering torn nets than others because mosquitoes were not collected outside the nets for possible comparison. The abundance of *An. gambiae s.l* reflected the selection of study locations with high vector potential. Assuming a sporozotic rate compared to other species, the high density of *An. gambiae s.l* may be due to its high anthropophilic and endophilic behavior [34,35]. *An. gambiae s.l* density in the torn nets varies between the climatic areas. Changes in temperature may have influenced the variability of mosquito density [36,37]. This study predicts high malaria vectors bite risk with torn nets in areas of permanent vector breeding sites.

We chose to use damaged, but otherwise new, LLINs rather than ordinary household nets. This aimed to measure protection level of chemical barrier provided by torn LLINs against resistant mosquitoes. At a pHI of 276, significant reduction of mosquito density was obtained with torn LLINs regardless of collection area. Due to the presence of chemical barrier, a low number of mosquitoes were able to enter torn LLINs. In another study [38] conducted indoor at the same location (Guinean site), in the same period, using human landing catch, an average of 20 bites /man/night was observed with *An. gambiae s.l*. These results indicated that chemical barrier of torn LLINs provided additional protection against mosquito’s biting ability in LLINs over that of non-insecticidal nets. While a reduction in vector density was observed when LLINs were compared with non-insecticidal nets, man-vector contact risk cannot be discounted. In fact, an average of 5 (3–7) *An. gambiae s.l*/man/nights can enter the torn LLINs to bite the sleeper. This show evidence that the sleepers are exposed to bites from malaria vectors when the nets are torn. With large holes (pHI > 276), *An. gambiae s.l* density could increase in the torn LLINs and protection of humans against mosquito bites could be lost when torn mosquito nets are used. Therefore, categorizing torn ITNs with a pHI over a cut-off of 88 as “ineffective” as done by Mutuku *et al.*[18] could be useful.

Asidi *et al.*[17] have also reported loss of protection of torn mosquito nets in southern Benin (resistance area). They treated torn ordinary households nets to show protection loss with resistant *An. gambiae* and observed, in 2008, low *Kdr* frequency (0.10) in the susceptible area (Sudanian site). High *Kdr* frequency (0.66) was observed in 2010 with this study. This may be due to the rapid spread of resistant mosquitoes and confirms the significant increase of *Kdr* frequency observed in Malanville from October 2008 to June 2010 by Djègbè et *al.*[39]. No significant reduction was observed between resistant *An. gambiae s.l* which can enter treated and non-insecticidal nets. The results show the possibility of resistant mosquitoes to penetrate both treated and non-insecticidal mosquito nets. This observation suggests that *Kdr* may be associated with higher than expected survival across the insecticidal barrier, compromising its protective effect, and rendering LLINs similar to non-insecticidal nets.

In Africa, current malaria control strategy is based on mass distribution of LLINs [40,41]. This technology is developed to reduce man-vector contact [42]. With physical damage of LLINs occurring rapidly [16,18,43], our study emphasizes the potential importance of care and repair of holes in LLINs. Repairing torn LLINs will not have the sole advantage to reduce vector contact but could also increase the operational life of LLINs.

Although the significant findings of this study, it had several limitations. Further assessment would have been possible if intact, old and worn out LLINs were included in the study design. Mosquito collections were restricted to new torn LLINs and limited to the assumption that intact mosquito nets do not allow mosquito entry as shown by Curtis *et al.*[44], Lines *et al.*[45] and a study on malaria in children sleeping under mosquito nets which were either intact or torn [46]. It is therefore important to conduct another study whenever possible to provide full details on the influence of holes and washing to the LLINs in preventing mosquito bites effect. Mosquito collections were also limited to environment with high vector breeding sites and the observed results could vary in other environments. In semi-arid or arid environment with low mosquito breeding sites for example, man-vector could be much reduced or prevented because of low vector density.

## Conclusions

At a pHI of 276, several mosquito species were able to enter the LLINs through the holes to bite. *An. gambiae s.l*, the main malaria vector was the most collected mosquito species that entered the torn LLINs to bite. The insecticidal barrier of the LLINs only reduced vector entry into LLINs with holes. Resistant mosquitoes have opportunity to enter both treated and non-insecticidal (control) nets. This study represents an alert for malaria control programs to increase public awareness of LLINs holes repair as a relevant integral part of programs to enhance the effectiveness of the control of malaria.

## Competing interests

There are neither any financial competing interests nor any non-financial competing interests (political, personal, religious, ideological, academic, intellectual, commercial or any other) to declare in relation to this manuscript.

## Authors’ contributions

VG collected analyzed, interpreted data and wrote the manuscript. RA was responsible for field collection, identification, processing of mosquitoes and helped in drafting the manuscript. RA contributed to the design of the study, helped in drafting the manuscript and revised the manuscript. FO, AS, RO GG and RA helped in data analysis, in reviewing the manuscript and helped with the activities. MCA conceived and designed the study, supervised fields and laboratory procedures, and review the manuscripts. All authors have read and approved the manuscript.

## Pre-publication history

The pre-publication history for this paper can be accessed here:

http://www.biomedcentral.com/1471-2458/13/751/prepub
